# Gut Microbiota and Colorectal Cancer: Is Microbial Dysbiosis in Carcinogenesis an Emerging Risk Factor?

**DOI:** 10.7759/cureus.102283

**Published:** 2026-01-25

**Authors:** Olurotimi J Badero, Emmanuel S Meribole, Olutomiwa Omokore, Ibrahim O Quadri, Perelade Kingdom, Ogbuiyi-chima C Ifeanyichukwu, Samuel O Ogunnoiki, Precious M Samuel-Ogunnoiki, Olaitan Adeyoola, Bamikole Osibowale, Nkechi Chima-Ogbuiyi, Mariam O Buari, Juliet Umeh, Adetola Adeyemi

**Affiliations:** 1 Interventional Cardiology, Iwosan Lagoon Hospital, Lagos, NGA; 2 Interventional Cardiology, Cardiac Renal and Vascular Associates P.C, Jackson, USA; 3 Medicine and Surgery, Babcock University Teaching Hospital, Ilishan-Remo, NGA; 4 Medicine and Surgery, Federal Medical Centre, Abuja, NGA; 5 Internal Medicine, Babcock University Teaching Hospital, Ilishan-Remo, NGA; 6 Internal Medicine, Benjamin S. Carson College of Health and Medical Sciences, Ilishan-Remo, NGA; 7 Surgery, Babcock University, Ilishan-remo, NGA; 8 Surgery, Benjamin S. Carson (Snr) School of Medicine, Ilishan-Remo, NGA; 9 Medicine and Surgery, Babcock University Teaching Hospital, Ilishan-remo, NGA; 10 Internal Medicine, Benjamin S. Carson (Snr) School of Medicine, Ilishan-Remo, NGA; 11 Medicine, Babcock University, Ilishan-Remo, NGA; 12 Cardiology, Carribean Tristate Heart Institute, Port of Spain, TTO; 13 Nursing, Herzing University, Milwaukee, USA; 14 Medicine, Babcock University Teaching Hospital, Ilishan-Remo, NGA; 15 Surgery, Igbinedion University, Okada, NGA; 16 Medicine and Surgery, Lagos University Teaching Hospital, Ikeja, NGA

**Keywords:** colorectal cancer, diagnostic biomarkers, gut-microbiota, microbial dysbiosis, personalised medicine

## Abstract

The gut microbiome has emerged as a critical factor in colorectal cancer (CRC) development, offering significant potential for early diagnosis and novel approaches. Current screening methods like colonoscopy and faecal immunochemical tests (FIT), while effective, face limitations in accessibility and patient compliance.

Recent research has identified distinct microbial signatures associated with CRC, including elevated levels of *Fusobacterium nucleatum* and specific metabolic byproducts, which could serve as non-invasive diagnostic biomarkers. The integration of microbiome analysis with existing screening techniques shows promise for improving early detection rates, particularly in underserved populations.

Furthermore, advances in multi-omics technologies are revealing novel mechanistic insights into how gut dysbiosis contributes to CRC progression, opening new avenues for targeted therapies and personalised prevention strategies. However, significant challenges related to standardisation and clinical implementation must be addressed to realise the full potential of these microbiome-based approaches in routine CRC care.

## Introduction and background

The gastrointestinal tract has been found to represent one of the largest interfaces between the host and environmental factors, as well as antigens, in the human body, estimated to be between 250 and 400 m² [1]. Over a man's average lifetime, about 60 tonnes of food pass through the human gastrointestinal tract, accompanied by microorganisms from the environment that threaten gut integrity. The collection of different microorganisms, bacteria, archaea, and eukarya colonising the gastrointestinal tract is termed the ‘gut microbiota’ and has co-evolved with the host over thousands of years to form an intricate and mutually beneficial relationship [1]. Human beings harbour between 10 and 100 trillion microbial cells in a symbiotic relationship [2]. Research estimates suggest that the microbial cells in the body could be up to 10 times more than those of human cells and that there are over 1,000 different species of microorganisms making up the human microbiota [2].

The gut microbiota confer numerous benefits to the host by performing diverse functions, including maintenance of intestinal barrier integrity, energy extraction from dietary substrates, protection against pathogenic organisms, and, notably, modulation of host immune responses [3]. In addition, the microbiota play a central role in xenobiotic metabolism. During this process, gut microorganisms chemically modify a wide range of substances, including dietary compounds, pharmaceuticals, environmental pollutants, and pesticides [3]. Through these metabolic and regulatory activities, the gut microbiota exert a profound influence on human health and have therefore been described as the body’s 'forgotten organ'. However, alterations in the composition and function of the gut microbial community, referred to as dysbiosis, can disrupt these beneficial roles and contribute significantly to disease development [1].

Dysbiosis refers to any alterations in the intestinal microbial population, functional structure, metabolic features, the gut epithelial barrier, and local deposition patterns compared with the homeostatic balance of a healthy individual’s microbiota [4]. Dysbiosis might be classified into three types: a lack of beneficial bacteria, an abundance of pathogenic microorganisms, and a lack of diversity in microbial species, which may all arise concomitantly [5]. This has been associated with the pathogenesis of disease conditions such as inflammatory bowel disease (IBD), irritable bowel syndrome (IBS), coeliac disease, allergy, asthma, metabolic syndrome, cardiovascular disease, and obesity [6].

Colorectal cancer (CRC) is the third most common cancer globally, accounting for approximately 10% of all cancer cases, and the second leading cause of cancer-related mortality [7]. In 2020, more than 1.9 million new cases of CRC and over 930,000 deaths due to CRC were reported worldwide [7]. Twin and family studies suggest that genetic predisposition accounts for only approximately 5% of the CRC cases, while it was estimated that about 90% of gastric cancer and CRC may be attributed to diet and other lifestyle factors [8]. This is supported by findings that CRC prevalence is significantly higher in African Americans than in native Africans [9]. 

The role of the microbiome in the development and progression of CRC is becoming increasingly appreciated. For instance, most CRC patients exhibit reduced bacterial diversity and richness relative to healthy individuals [9]. Additionally, certain bacterial species, such as *Fusobacterium nucleatum*, *Streptococcus bovis*, and *Bacteroides fragilis*, have been linked to CRC, either by producing virulence factors or by producing pathogenic microbial metabolites [10]. This review highlights the role of dysbiosis in colorectal carcinogenesis and its recognition as an emerging risk factor.

## Review

Methodology

This narrative review synthesises current evidence on the role of gut dysbiosis in CRC, focusing on pathogenic mechanisms, diagnostic potential, and emerging clinical applications. A literature search was conducted using PubMed/MEDLINE, Scopus, Web of Science, and Google Scholar for English-language publications from January 2000 to June 2025. Search terms included combinations of "gut microbiome," "dysbiosis," "colorectal cancer," "microbial metabolites," "Fusobacterium nucleatum," "bile acids," "inflammation," "microbiome-based diagnostics," and "multi-omics." Peer-reviewed original studies, clinical and experimental research, and high-quality reviews relevant to CRC were included, while studies unrelated to gastrointestinal malignancy or lacking mechanistic or clinical relevance were excluded. Additional sources were identified through reference list screening of key articles. As a narrative review, no formal risk-of-bias assessment or meta-analysis was performed; instead, studies were selected and thematically integrated based on biological plausibility, consistency of findings, and translational relevance to CRC pathogenesis, screening, and precision prevention.

Gut microbial ecosystem and functions

Humans exist in a complex symbiotic relationship with trillions of microorganisms, including bacteria, viruses, fungi, archaea, and bacteriophages, collectively termed the microbiota. These microorganisms colonise multiple anatomical sites such as the skin, oral cavity, airways, genitourinary tract, and gastrointestinal tract. While fungal and viral components of the microbiome have received increasing attention, the majority of research has focused on bacterial populations, particularly those residing in the gut, due to their abundance, diversity, and profound influence on host physiology [11,12].

The intestinal microbiota is the most diverse and variable microbial ecosystem in the human body. It is estimated that approximately 3,000 bacterial species inhabit the human gastrointestinal tract, classified into 11 distinct phyla. Among these, Firmicutes and Bacteroidetes account for nearly 90% of the gut microbial population, with Actinobacteria, Proteobacteria, and Verrucomicrobia constituting smaller but functionally important proportions [12,13]. Compared to microbiomes at other anatomical sites, gut microbial composition exhibits greater inter-individual variability, influenced by host genetics, environmental exposure, diet, medication use, and lifestyle factors.

The establishment of the gut microbiome begins early in life. Evidence suggests that intrauterine exposure to bacteria may contribute to the foundational microbial landscape of the foetus. Following birth, multiple exposures further shape microbial development, including maternal microbiota, mode of delivery, antibiotic exposure, geography, and feeding practices such as breastfeeding or formula feeding [13]. In infancy, the gut microbiome is dominated by human milk oligosaccharide-metabolising bacteria, particularly Bifidobacterium species within the Actinobacteria phylum. With the introduction of solid foods, microbial composition shifts toward an adult-like profile enriched with *Bacteroides* species, which metabolise complex carbohydrates such as starches [12,13]. In healthy adults, Firmicutes and Bacteroidetes typically exist in relatively balanced proportions, and deviations from this equilibrium are associated with pathological states, including inflammation, infection, and obesity [12].

The gut microbiome performs essential functions that extend beyond digestion, influencing host metabolism, immune regulation, and central nervous system (CNS) activity [13]. Metabolically, intestinal bacteria contribute to vitamin synthesis, detoxification of carcinogens, fermentation of non-digestible dietary fibres such as pectin and mannans, metabolism of orally administered drugs, and the synthesis and transformation of bile acids [13]. Through these mechanisms, the microbiota acts as a metabolic organ that complements host physiology.

From an immunological perspective, the gut microbiota is essential for maintaining the integrity of the intestinal epithelial barrier (IEB), which regulates nutrient absorption while preventing translocation of pathogens and toxins. One of the most important microbial products involved in immune modulation is short-chain fatty acids (SCFAs), including acetate, butyrate, and propionate, generated through bacterial fermentation of dietary fibres [14]. Butyrate serves as a primary energy source for colonocytes and enhances mucosal barrier integrity by upregulating tight junction proteins, while acetate and propionate participate in hepatic lipogenesis and gluconeogenesis [11,14]. These SCFAs also modulate immune responses by interacting with host immune cells and influencing cytokine production.

The gut microbiome further exerts influence over the CNS via the microbiota-gut-brain axis (MGBA), a bidirectional communication network involving the enteric nervous system, vagus nerve, immune system, and circulatory pathways [15]. Microbiota-derived metabolites, neuroactive compounds, and gut hormones regulate neurotransmitter synthesis and neuropeptide release, thereby modulating brain function, behaviour, and neuroinflammation. This axis plays a protective role against inflammation associated with ageing and neurodegenerative disease [15].

Balanced microbiota versus dysbiosis

Under physiological conditions, the gut microbiota exists in a dynamic but stable state of homeostasis with the host. This balanced ecosystem comprises a diverse and functionally complementary microbial community that supports metabolic efficiency, immune tolerance, and epithelial integrity, thereby reducing susceptibility to disease [13]. However, this equilibrium is highly sensitive to perturbation. Alterations in microbial composition and function, collectively referred to as dysbiosis, can disrupt host-microbiota symbiosis and predispose individuals to disease [11,13].

Dysbiosis is characterised by a loss of beneficial commensal bacteria, overgrowth of potentially pathogenic microorganisms, and reduced microbial diversity [16]. While the gut microbiota exhibits substantial resilience, single disturbances are often insufficient to induce lasting dysbiosis. Instead, a combination of host-specific factors (genetics, ageing, health status, lifestyle) and environmental influences (diet, medications, xenobiotics, hygiene) typically drives pathological shifts [17].

Diet is one of the most influential determinants of gut microbial composition, capable of inducing rapid and profound changes. Diets rich in food additives and preservatives promote the expansion of Proteobacteria, a recognised hallmark of dysbiosis, leading to mucosal inflammation and compromise of the epithelial barrier [16,17]. Similarly, oral medications exert significant effects on gut microbial communities. Antibiotics disrupt microbial diversity and enable opportunistic pathogens to proliferate, while non-antibiotic drugs such as fluoxetine, nonsteroidal anti-inflammatory drugs (NSAIDs), and proton pump inhibitors (PPIs) have also been shown to alter microbial composition [18].

Host-specific factors further contribute to dysbiosis. Ageing is associated with reduced microbial diversity and chronic low-grade inflammation, impairing digestion, nutrient absorption, and immune function. Genetic variation also influences microbial composition, affecting susceptibility to dysbiosis-related diseases [18].

Chronic dysbiosis compromises immune homeostasis by disrupting interactions between commensal microbes and host immune cells. In healthy individuals, microbial components such as *Bacteroides* polysaccharides and segmented filamentous bacteria stimulate Th1 and Th17 cell responses, facilitating clearance of translocated bacteria. Dysbiosis weakens this defence, increasing bacterial translocation across the epithelium and activating toll-like receptors (TLRs), which trigger excessive pro-inflammatory cytokine production and epithelial damage [17].

The maturation of innate immunity is also dependent on microbial exposure. Dysbiosis impairs neutrophil and dendritic cell function, reducing secretion of type I interferons and interleukin-15 [17]. Furthermore, the absence of normal microbiota delays myeloid cell development in the bone marrow, increasing susceptibility to infection and allergic disease. Epidemiological studies have linked early-life antibiotic exposure to increased risk of IBD, asthma, and atopic dermatitis later in life [17].

Metabolic disorders, including obesity and type 2 diabetes mellitus (T2DM), are strongly associated with dysbiosis. Proposed mechanisms include reduced SCFA-producing bacteria, impaired peroxisome proliferator-activated receptor gamma activity, expansion of pathogenic facultative anaerobes, diminished T-cell function, and chronic inflammation [19]. Consequently, dysbiosis-associated inflammation contributes to a wide spectrum of diseases, including obesity, asthma, necrotising enterocolitis, IBD, Alzheimer’s disease, rheumatoid arthritis, colon cancer, and other malignancies [18].

Does dysbiosis cause CRC, or is it correlated?

The role of the gut microbiome in CRC has emerged as a rapidly expanding area of research. Although numerous studies demonstrate a strong association between dysbiosis and CRC [20,21], the directionality of this relationship remains unresolved. It is unclear whether dysbiosis initiates colorectal carcinogenesis or whether tumour development itself drives microbial alterations.

Longitudinal cohort studies, such as the COLON study, are essential to clarify this relationship by tracking microbiome changes over time and correlating them with the development of precancerous lesions and CRC [22]. Experimental models further support causality. Germ-free mouse models, considered the gold standard for microbiome research and faecal microbiota transplantation (FMT) studies, allow direct investigation of microbial influence on tumour development [23]. Wong et al. demonstrated that germ-free mice colonised with faecal samples from CRC patients developed significantly more dysplasia and macroscopic polyps than those receiving samples from healthy controls [24]. Mechanistic studies have identified microbial metabolites, toxins, and virulence factors that promote carcinogenesis through inflammation, DNA damage, and oncogenic signalling [25]. However, interpretation is complicated by inter-individual variability in microbiome composition, diet, antibiotic exposure, and other confounders.

Clinical evidence highlights complex interactions between microbial factors and host genetics. For example, the tumorigenic effects of *F. nucleatum* vary according to microsatellite instability (MSI) status, with high-MSI tumours exhibiting enhanced immune responses and low-MSI tumours demonstrating immune suppression mediated by microbial inflammation [26].

Chronic inflammation is a recognised driver of cancer progression. Dysbiotic microbiota promote persistent inflammatory signalling through cytokines such as IL-1, IL-6, IL-10, and tumour necrosis factor-alpha, activating NF-κB, Wnt, and MAPK pathways, inhibiting apoptosis, and increasing oxidative stress [27]. Microbial toxins such as colibactin and *B. fragilis* toxin (BFT) induce DNA damage and double-strand breaks, supporting a 'two-hit' model of carcinogenesis whereby genetic mutations are compounded by sustained microbial-driven inflammation [28].

Altered bile acid metabolism further contributes to CRC pathogenesis. Primary bile acids synthesised in the liver are converted by gut microbiota into secondary bile acids such as deoxycholic acid (DCA) and lithocholic acid (LCA). DCA promotes DNA damage, activates oncogenic signalling pathways, and suppresses tumour suppressor p53 activity [10]. Elevated faecal DCA levels have been observed in populations with high CRC incidence, particularly those consuming high-fat, low-fibre diets. Animal models demonstrate that DCA alone can induce tumour formation, underscoring its carcinogenic potential.

Disruption of gut barrier integrity, or 'leaky gut,' also plays a critical role. High-fat diet-induced dysbiosis increases intestinal permeability, facilitating translocation of lipopolysaccharides and promoting systemic inflammation [29]. Bacterial toxins from Escherichia coli and *B. fragilis* directly damage epithelial cells through diverse mechanisms, including inhibition of protein synthesis, disruption of tight junctions, and activation of pro-inflammatory and oncogenic pathways [30-32].

Clinical applications of gut dysbiosis in CRC

Understanding the relationship between dysbiosis and CRC has important clinical implications for prevention, diagnosis, prognosis, and treatment [33]. Identifying dysbiosis as a modifiable risk factor offers opportunities for dietary interventions and microbiota-targeted strategies to reduce CRC incidence. The gut microbiome significantly influences responses to cancer therapy, particularly immunotherapy. Optimal responses to immune checkpoint inhibitors depend on an intact commensal microbiota [33-36]. Increased abundance of Bacteroidetes has been associated with improved response to ipilimumab, a CTLA-4 inhibitor [37,38]. Emerging evidence suggests that specific microbial species and metabolites modulate treatment efficacy in CRC [39].

Microbiota modulation may also reduce chemoresistance. Dietary fibre increases SCFA-producing bacteria such as Prevotella, improving glucose metabolism and reducing glycolysis-dependent drug resistance mechanisms [40]. Short-chain fatty acids like butyrate reduce inflammation, promote apoptosis, strengthen the epithelial barrier, and slow CRC progression [40,41].

Microbiome-based diagnostics represent a promising frontier. Current non-invasive tests, such as faecal immunochemical testing (FIT) and faecal micro-ribonucleic acids (miRNAs), demonstrate limited sensitivity for early-stage CRC [42,43]. Incorporation of microbial biomarkers could significantly enhance screening accuracy, particularly for early-onset CRC, which is rising among younger populations without genetic predisposition [44,45].

Studies have identified CRC-specific microbial signatures, including elevated *F. nucleatum* and *Peptostreptococcus anaerobius* levels, with potential application in stool-based screening [46,47]. Combining microbiome analysis with FIT improves sensitivity and reduces false positives, potentially decreasing unnecessary colonoscopies [48-51]. Multi-omics approaches integrating metagenomics, proteomics, and metabolomics are likely to become standard tools for CRC detection and risk stratification [52-57].

The evolving role of gut dysbiosis in CRC

The understanding of CRC is undergoing a paradigm shift as growing evidence demonstrates that gut dysbiosis plays an active role in carcinogenesis rather than being a passive consequence of disease. Microbial communities within the gastrointestinal tract contribute to inflammation, metabolic dysregulation, and tumour development, creating new opportunities for early detection and precision intervention [58].

Conventional CRC screening methods have notable limitations. Colonoscopy, while effective, is invasive and costly, limiting accessibility in low-resource settings, while FIT and blood-based biomarkers such as carcinoembryonic antigen (CEA) and Septin9 lack sufficient sensitivity for early-stage disease detection [59-61]. In contrast, gut microbiome profiling has emerged as a promising non-invasive diagnostic tool. Studies have consistently identified CRC-associated microbial signatures, including elevated levels of *F. nucleatum*, enterotoxigenic *B. fragilis*, and *P. anaerobius*, which show strong correlations with inflammation and tumour development [49,62-65].

Beyond diagnostics, microbial metabolites such as secondary bile acids may facilitate the detection of early-stage CRC, and combining microbiome analysis with FIT improves screening sensitivity [64,66]. Integration of microbiomics with metagenomics and metabolomics offers novel avenues for personalised prevention and therapy. However, large-scale validation, standardisation, and ethical considerations remain critical before widespread clinical adoption [67].

## Conclusions

The study of gut dysbiosis in CRC represents a frontier in oncological research, with far-reaching implications for clinical practice. Microbial biomarkers and metabolites have demonstrated robust associations with CRC, paving the way for minimally invasive screening tools that could complement or surpass existing methods. The integration of multidisciplinary data not only deepens our understanding of host-microbe interactions in carcinogenesis but also fuels innovative therapeutic strategies, from microbiota modulation to personalised immunotherapy. However, the translation of these discoveries into widespread clinical use demands concerted efforts, such as large-scale validation studies, standardised analytical frameworks, and policies to ensure global accessibility. As this field advances, the vision of precision CRC prevention guided by an individual's microbial and molecular profile moves closer to reality. By harnessing the gut microbiome’s diagnostic and therapeutic potential, we may ultimately reduce the global burden of CRC through earlier detection, targeted prevention, and more effective treatments.
